# Polycystic Ovary Syndrome and Cardiometabolic Health Across Generation: Insights from 25 Years of the Tehran Lipid and Glucose Study (TLGS)

**DOI:** 10.5812/ijem-167151

**Published:** 2026-01-31

**Authors:** Mahsa Noroozzadeh, Marzieh Saei Ghare Naz, Maryam Mousavi, Mahbanoo Farhadi-Azar, Maryam Farahmand, Shabahang Amirshekari, Fereidoun Azizi, Fahimeh Ramezani Tehrani

**Affiliations:** 1 Reproductive Endocrinology Research Center, Research Institute for Endocrine Molecular Biology, Research Institute for Endocrine Sciences, Shahid Beheshti University of Medical Sciences, Tehran, Iran; 2 Endocrine Research Center, Research Institute for Endocrine Disorders, Research Institute for Endocrine Sciences, Shahid Beheshti University of Medical Sciences, Tehran, Iran; 3 Foundation for Research & Education Excellence, Vestavia Hills, AL, USA

**Keywords:** Polycystic Ovary Syndrome, Cardiometabolic Disorders, TLGS, Offspring

## Abstract

**Context:**

Polycystic ovary syndrome (PCOS) affects an increasing number of reproductive-aged women and has been progressively associated with a spectrum of metabolic disturbances. Understanding its long-term cardiometabolic consequences, both in affected women and in their offspring, is critical for informing early prevention and targeted intervention strategies. This study provides a comprehensive synthesis of published data from the Tehran lipid and glucose study (TLGS) on women with PCOS, offering insights into epidemiological patterns, risk factors, and potential intergenerational impacts in this population.

**Evidence Acquisition:**

This narrative review provides a comprehensive summary of TLGS-based publications addressing cardiometabolic disorders associated with PCOS. Relevant studies were identified through a comprehensive search of PubMed (including Medline), Web of Science, and Scopus.

**Results:**

Data from the TLGS indicate a rising prevalence of PCOS over approximately a decade, increasing from 8.5% based on the National Institutes of Health (NIH) criteria to 13.6% (NIH), 17.8% (androgen excess (AE)-PCOS Society), and 19.4% (Rotterdam criteria). Women with PCOS in early adulthood demonstrated elevated levels of insulin resistance (IR) as reflected by higher HOMA-IR and fasting insulin values, and subsequently experienced a heightened likelihood of developing type 2 diabetes mellitus (T2DM), hypertension, metabolic syndrome (MetS), and obesity, with adjusted hazard ratios ranging from approximately 0.58 to 2.08 across multiple publications. Notably, the prevalence of certain cardiometabolic disorders appeared to decline after the age of 40 years. Longitudinal data showed that trajectories of HOMA-IR and fasting insulin differed depending on obesity status in women with PCOS, and that cardiometabolic risk burden varied across diagnostic phenotypes. Interestingly, PCOS was not associated with an increased risk of silent coronary artery disease. Nevertheless, the Framingham risk score (FRS) showed good predictive performance for cardiovascular disease (CVD) in the PCOS population, with a 38% increase in CVD risk per 1-unit increment in FRS among affected women. Intergenerational analyses further highlighted that the offspring of women with PCOS carry a higher predisposition to metabolic disorders. Daughters of affected women were found to have increased risks of T2DM, overweight, and MetS. At the same time, sons exhibited significantly elevated risk of pre-diabetes mellitus, with adjusted hazard ratios ranging from approximately 1.3 to 2.4. These findings underscore both the individual and transgenerational cardiometabolic disorders of PCOS.

**Conclusions:**

Publications from the TLGS provide consistent evidence that PCOS is associated with a spectrum of early cardiometabolic disturbances in affected women with potential adverse impacts extending to their offspring. These observations underscore the importance of implementing age-specific metabolic screening and preventive interventions for women with PCOS and their offspring. Furthermore, they highlight the need for long-term, prospective follow-up studies to elucidate underlying causal pathways and identify targeted strategies for timely intervention.

## 1. Context

Polycystic ovary syndrome (PCOS) is the most frequently diagnosed endocrine disorder affecting reproductive-aged women, occurring in approximately 10% of women (1, 2). It is characterized by chronic oligo-anovulation, hyperandrogenism, and polycystic ovaries. Alongside its effects on reproductive health, PCOS is often associated with numerous cardiometabolic disorders, such as obesity, type 2 diabetes mellitus (T2DM), abnormal lipid profile (dyslipidemia), metabolic syndrome (MetS), and hypertension, all of which may increase the risk of cardiovascular diseases (CVDs) in PCOS patients (3). Evidence revealed that PCOS not only affects different health aspects of women but also threatens their offspring’s health (4-6). Cardiometabolic disorders linked to PCOS have primarily been investigated through data derived from clinical settings or case-control studies. However, these research methodologies present several limitations, including potential selection bias, limited generalizability, and inability to establish temporal relationships. There is a limited body of population-based research that has systematically examined the cardiometabolic characteristics and risks associated with PCOS (7-9). These studies are relatively scarce compared to those conducted in clinical or controlled environments, which restricts the depth of understanding regarding the prevalence, progression, and long-term outcomes of cardiometabolic complications in the broader population of women with PCOS. Moreover, none of these studies have employed a family-based design, which limits the understanding of the potential intergenerational effects of PCOS. As a result, the impact of PCOS on the health and cardiometabolic risk profiles of subsequent generations remains poorly characterized. Investigating these familial patterns through longitudinal, family-based research is crucial to elucidate the hereditary and environmental influences of PCOS and to develop targeted prevention and management strategies for affected families.

The Tehran lipid and glucose study (TLGS) is a unique, highly reliable longitudinal dataset focused on cardiometabolic disorders in an urban West Asian population (10, 11). This study utilizes rigorously validated assessment methods and protocols, including the administration of detailed structured questionnaires, comprehensive physical examinations, meticulous verification of participants’ prior medical histories and hospital records, and standardized biochemical and hormonal evaluations. These assessments are conducted within a familial context, allowing for the simultaneous evaluation of women and their biological relatives. As a large-scale, population-based cohort, the TLGS represents an invaluable resource for investigating critical gaps in understanding cardiometabolic health, particularly among women diagnosed with PCOS and their offspring, thereby enabling the exploration of familial and intergenerational health influences. Given that TLGS is a unique cohort with over two decades of follow-up in a population representative of the capital city of Iran, it can offer an important overview of PCOS and its impact on women’s and family health in the Iranian population. No other Iranian study provides comparable long-term data.

## 2. Evidence Acquisition

This narrative review consolidated findings from all English-language publications related to cardiometabolic disorders in women with PCOS and their offspring (daughters and sons) in TLGS with a quarter-century follow-up. To achieve this, an extensive literature search was performed across PubMed, Web of Science, and Scopus databases to identify relevant studies. The search was conducted using the following MeSH terms: “Body Mass Index”, Obesity, Overweight, Pre-DM, “Prediabetes Mellitus”, Prediabetes, “Metabolic disorders”, T2DM, “Type 2 diabetes mellitus”, “Fasting blood glucose”, Hyperglycemia, Hypoglycemia, Insulin, “Insulin resistance”, “Insulin sensitivity”, HOMA-IR (Homeostatic Model Assessment for Insulin Resistance), “Glucose tolerance”, Triglyceride, Cholesterol, HDL, LDL, Dyslipidemia, “Blood pressure”, Pre-Hypertension, Hypertension, “Waist circumference”, “Metabolic syndrome”, “Cardiovascular diseases”, CVDs, “Cardiometabolic disorders” AND “Polycystic ovary syndrome” AND “Tehran lipid and glucose study”.

Eligibility: All peer-reviewed original published articles in the frame of TLGS in which: (1) Report PCOS as the exposure, and (2) report at least one cardiometabolic disorder (outcome) in women or their offspring, were included.

Table 1 summarizes the details of the study protocol including interviews (questionnaire), physical examinations, biochemical and hormonal assessments, ultrasonography (ultrasound), and definitions.

**Table 1.** Data Collection Protocol for the Study

Data Collection	Process
**Questionnaire**	A standard questionnaire covering demographic data and reproductive history, focusing on menstrual cycle regularity, gynecological history, and hyperandrogenic manifestations, was administered.
**Physical examinations**	Examination for clinical evidence of hirsutism, acne, alopecia, acanthosis nigricans, and other signs of virilism was performed. Hirsutism was evaluated using the modified Ferriman-Gallwey (mFG) scoring method (12) by a general practitioner who had completed a one-month observational training at a PCOS clinic under the supervision of a single endocrinologist. Acne was evaluated according to its number, type, and anatomical distribution. Androgenic alopecia was defined as moderate to severe hair loss at the temples or widespread thinning across the crown (13-16). Acanthosis nigricans (14)
**Blood sampling for biochemical and hormonal assessments**	A fasting venous blood sample was collected in the morning from participants on the second or third day of either their spontaneous or progesterone-induced menstrual cycle. Levels of DHEAS, 17-OH-P, TT, and A4 were measured using EIA (Diagnostic Biochem Canada Co., Ontario, Canada). SHBG levels were determined using an IEMA (Mercodia, Uppsala, Sweden). All ELISA were carried out with the Sunrise ELISA reader. (Tecan Co., Salzburg, Austria). LH, FSH, PRL, and TSH concentrations were determined using an IRMA technique (Izotop, Budapest, Hungary) using a gamma counter (Wallac Wizard, Turku, Finland). All assays demonstrated intra- and inter-assay variability coefficients of less than 6.5%. All sera were maintained at -80°C until laboratory assessments were performed.
**Ultrasonography (ultrasound)**	Ovarian ultrasound assessments, both transvaginal and transabdominal, were performed on study participants utilizing a 3.5-MHz transducer for the abdominal approach and a 5-MHz transducer for the vaginal approach. An experienced sonographer conducted examinations using both ultrasound devices. Virgin patients underwent a transabdominal ultrasound examination. The ultrasound assessments were carried out concurrently with the blood sampling. The high-quality portable ultrasound system used for these examinations was the SONOACE R3 (Product name: USS-SAR3N2R/WR).
**Definitions **	In this study, PCOS was diagnosed based on the Rotterdam criteria, requiring the presence of at least 2 of the following criteria: (1) HA was identified based on the presence of CH and/or BH. (A) CH was defined by the presence of hirsutism (mF-G score ≥ 8), acne, or signs of androgenic alopecia. (B) BH was defined as elevated levels of TT and/or FAI and/or DHEAS and/or A4, above the upper 95th percentile, in women not received hormonal therapy and without clinical signs of hyperandrogenism or menstrual irregularities (TT = 0.89 ng/mL, A4 = 2.9 ng/mL, DHEAS = 179 μg/dL, FAI = 5.39). (2) Oligo-anovulation was defined as a self-reported menstrual cycle length of 35 days or longer, or fewer than 10 menstrual periods per year. (3) Polycystic ovarian morphology was defined based on the presence of twelve or more follicles (each measuring 2 - 9 mm in diameter) in the whole ovary and/or an ovarian volume ≥ 10 mL. In our study, PCOS was diagnosed according to the NIH criteria, requiring the coexistence of oligo/anovulation and either CH or BH. PCOS, according to the AE PCOS Society was defined as CH or BH and oligo/anovulation or polycystic ovaries on ultrasound. PCOS according to the Rotterdam criteria was defined as at least 2 of 3 criteria.T2DM, hypertension, MetS, Pre-DM, dyslipidemia, obesity, CKD, liver function, and CVDs were defined according to standard protocol of TLGS (8, 10, 17, 18).

Abbreviation: AE, androgen excess; DHEAS, dehydroepiandrosterone sulfate; 17-OH-P, 17-hydroxyprogesterone; TT, total testosterone; A4, androstenedione; EIA, enzyme immunoassay; SHBG, Sex Hormone-Binding Globulin; IEMA, immunoenzymometric assay; ELISA, enzyme-linked immunosorbent assays; LH, Luteinizing hormone; FSH, follicle-stimulating hormone; PRL, prolactin; TSH, thyroid-stimulating hormone; IRMA, immunoradiometric assay; HA, Hyperandrogenism; CH, clinical hyperandrogenism; BH, biochemical hyperandrogenemia; FAI, Free Androgen Index; Pre-DM, Prediabetes mellitus; CKD, chronic kidney disease.

## 3. Results

Finally, 18 relevant articles were included in this review, comprising 7 cross-sectional and 11 cohort studies. All publications were conducted among women with a well-defined diagnosis of PCOS within the reproductive age range of 18 to 49 years. The timeline for the publications derived from the TLGS begins in 2011 and extends through 2025. Notably, research focusing on the cardiometabolic health of both male and female offspring of women with PCOS has emerged recently, with the earliest studies appearing from May 2022 onward.

### 3.1. Prevalence of Polycystic Ovary Syndrome

**Figure 1.**
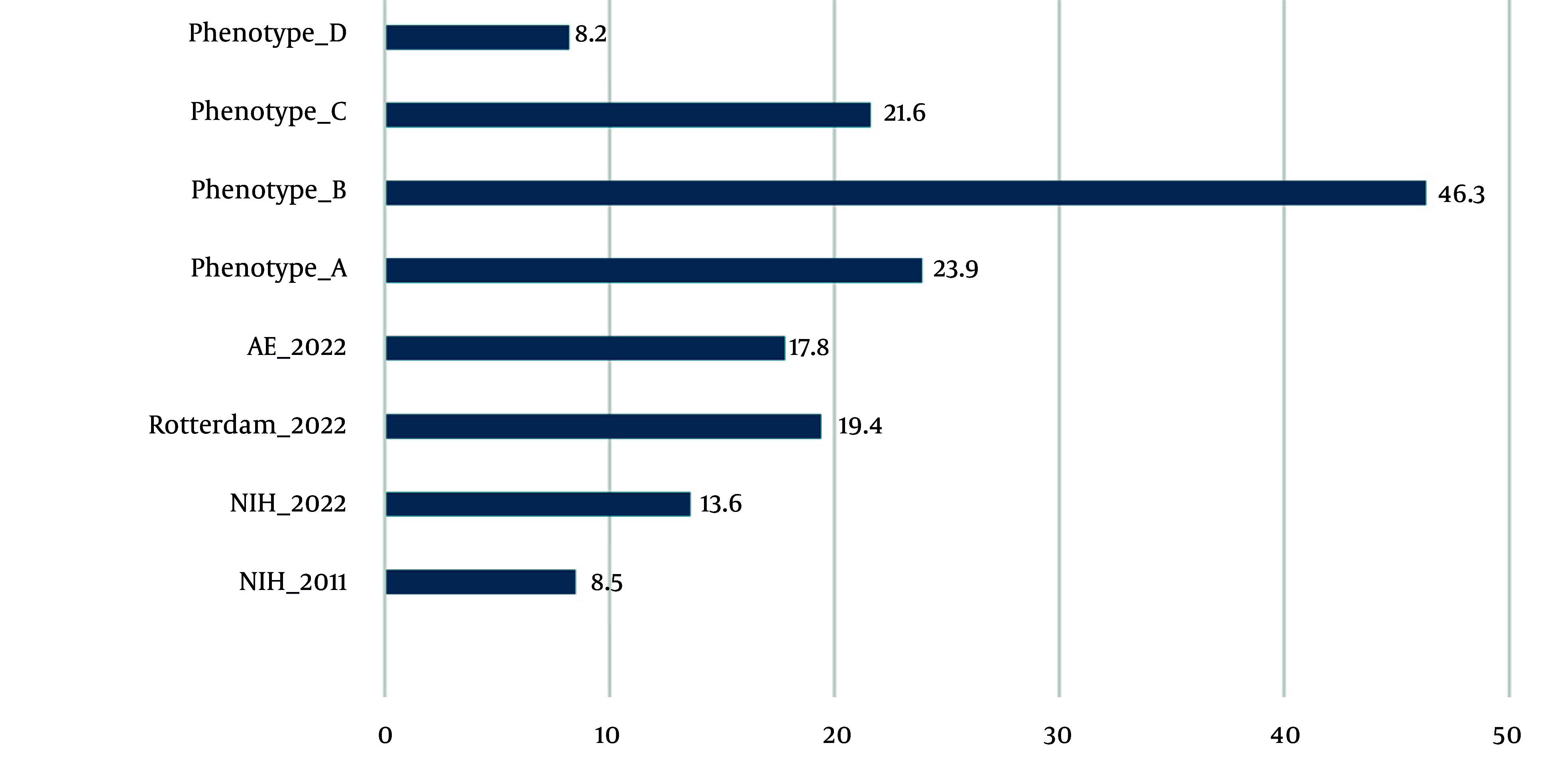
Prevalence of polycystic ovary syndrome (PCOS) (Abbreviations: AE, androgen excess; NIH, National Institutes of Health)

Initially, within the TLGS, PCOS was defined according to the NIH criteria. From the third phase of TLGS onward, the diagnostic definition was updated to the Rotterdam criteria. Early cohort evaluations based on the NIH criteria documented a PCOS prevalence of 8.5% (95% CI: 6.8 - 10.2) (19). Subsequent analyses conducted approximately one decade later reported the prevalence of PCOS according to different diagnostic criteria as follows: 13.6% based on the NIH criteria; 19.4% according to the Rotterdam criteria, with the most common phenotypes being A (23.9%) and B (46.3%); and 17.8% using the AE-PCOS Society criteria (20). Figure 1 and Table 2 show the prevalence of PCOS among women participating in the TLGS over time.

**Table 2.** Prevalence of Polycystic Ovary Syndrome and Cardiometabolic Disorders in Women with Polycystic Ovary Syndrome and Their Offspring in Tehran Lipid and Glucose Study

Parameters/Author (y) and Ref. No.	Sample Size	Main Findings
**Prevalence of PCOS**		
Tehrani et al. (2011) (19)	n = 1,002	-Prevalence of PCOS: 8.5% (NIH criteria)-Prevalence of PCOS: 13.6% (NIH criteria)-Prevalence of PCOS: 19.4% (Rotterdam criteria)-Prevalence of PCOS: 17.8% (AE-PCOS Society criteria)
Farhadi-Azar et al. (2022) (20)	n = 1,960	
**Glucose disturbances**		
Ramezani Tehrani et al. (2015) (7)	PCOS: n = 85, non-PCOS (controls): n = 552	-Mean of insulin (3.55, CI: 0.66 - 6.45), HOMA-IR (0.63, CI: 0.08 - 1.18), and HOMA-β (45.90, CI: 0.86 - 90.93) were significantly higher in PCOS than in healthy women. -In longitudinal comparison, insulin and HOMA-IR increased by 6.7% and 14.6%, respectively, in controls, and decreased by 10.6% and 5%, respectively, in PCOS women.
Behboudi-Gandevani et al. (2016) (21)	PCOS: n = 50, eumenorrheic non-hirsute women: n = 704	- Higher prevalence of IR in PCOS women compared to those without the condition (34% vs. 26%).
Kazemi Jaliseh et al. (2017) (9)	PCOS: n = 178, non PCOS: n = 1,524	- Incidence rates of both T2DM and pre-DM were significantly higher in PCOS women compared to non-PCOS women (13.4 vs. 4.2) and (30.3 vs. 23.9), respectively.
Ramezani Tehrani et al. (2025) (22)	PCOS: n = 287, isolated PCOS: n = 536, non-PCOS: n = 936	-Obese PCOS women had a significant longitudinal increase in IR in comparison to those without obesity. -PCOS women without obesity demonstrated an improvement in IR over time.
Noroozzadeh et al. (2022) (23)	Daughters of women with PCOS: n = 211, daughters of non-PCOS women: n = 757	- Significant increased risk of developing T2DM in daughters of PCOS women (HR: 2.44; 95% CI, 1.13 - 5.27) compared to daughters of non-PCOS women.
Farhadi-Azar et al. (2023) (24)	Sons of women with PCOS: n = 409, sons of non-PCOS: women n = 954	- Significant higher risk of pre-DM among the sons of PCOS women (HR: 1.46; 95% CI: 1.20 - 1.78) compared to sons of non-PCOS women.
**MetS and components**		
Behboudi-Gandevani et al. (2016) (21)	PCOS: n = 50, eumenorrheic non-hirsute women: n = 704	-No statistically significant difference in MetS prevalence between PCOS and non-PCOS women (15% vs. 14%)
Behboudi-Gandevani et al. (2018) (8)	PCOS: n = 178, non-PCOS (controls): n = 1,524	Significant differences in the incidence rates of MetS (21 vs. 22.7) and dyslipidemia (46.1 vs. 46) between PCOS and non-PCOS women. -PCOS women age ≤ 40 exhibited a higher risk of MetS (HR: 1.81; 95% CI: 1.1 - 2.9). -The risk of dyslipidemia did not differ significantly between PCOS and non-PCOS women.
Farhadi-Azar et al. (2022) (20)	n = 1,960	-A less favorable lipid profile in PCOS women (phenotypes A, B, C) compared to non-PCOS women. -Elevated rate of hypertriglyceridemia in PCOS women compared to non-PCOS women. -A significantly higher prevalence of MetS in PCOS women (phenotype A) compared to non-PCOS women.
Noroozzadeh et al. (2023) (25)	Daughters of women with PCOS: n = 323, daughters of non-PCOS women: n = 1,125	-Increased risk of developing MetS in daughters of PCOS women (HR: 1.34; 95% CI: 1.00 - 1.80) compared to daughters of non-PCOS women.
Noroozzadeh et al. (2024) (26)	Sons of women with PCOS: n = 523, sons of non-PCOS women: n = 1,390	-A higher risk of MetS was not observed in the sons of PCOS women compared to non-PCOS women.
**Hypertensive disorders and CVDs**		
Ramezani Tehrani et al. (2015) (7)	PCOS: n = 85, non-PCOS: n = 552	-No significant difference was detected in SBP and DBP values of PCOS women compared to non-PCOS women.
Behboudi-Gandevani et al. (2018) (8)	PCOS: n = 178, non-PCOS (controls): n = 1,524	-A higher risk of developing hypertension in PCOS women aged ≤ 40 years (HR: 2.08; 95% CI: 1.0 - 3.9) compared to non-PCOS women.
Mahboobifard et al. (2022) (27)	PCOS: n = 356, non-PCOS: n = 1,235	-No significant association between PCOS status and the risk of silent CAD (HR: 0.96; 95% CI: 0.86 - 1.08).- Regardless of PCOS status, women with a previous history of silent CAD had a 2.25-fold higher incidence of CVDs in later life compared to those without such a history (95% CI: 1.63 - 3.10). -The presence of PCOS was associated with a 42% reduction in CVDs incidence, regardless of silent CAD or conventional risk factors (HR: 0.58; 95% CI: 0.35 - 0.98).
Amiri et al. (2025) (28)	PCOS: n = 215	-In women with PCOS, each one-unit increase in the FRS was associated with a 38% rise in the risk of CVDs (HR: 1.38; 95% CI: 1.14 - 1.66).
**Adiposity**		
Ramezani Tehrani et al. (2015) (7)	PCOS: n = 85, non-PCOS: n = 552	-No significant difference in WC between PCOS and non-PCOS women.
Behboudi-Gandevani et al. (2016) (21)	PCOS: n = 50, eumenorrheic non-hirsute women: n = 704	-No significant difference in ABSI between PCOS and non-hirsute women [0.76 (0.05) vs. 0.76 (0.053)]. -The AUC (CI 95%) of WHtR for expecting IR and MetS in PCOS women exceeded 0.75, indicating good predictive performance.
Ehsani et al. (2016) (29)	PCOS: n = 53, normo-ovulatory women: n = 167	-A markedly higher prevalence of cardiometabolic disturbances in PCOS women with VAD compared to those without VAD.
Behboudi-Gandevani et al. (2018) (8)	PCOS: n = 178, non-PCOS (controls): n = 1,524	-The incidence rate of obesity in PCOS and non-PCOS women was 22.6 (95% CI: 15.5-33.0) and 24.0 (95% CI: 21.3 - 27.0), respectively. -The risk of central and general obesity increased in PCOS women compared to non-PCOS women.
Farhadi-Azar et al. (2022) (20)	n = 1,960	-Adiposity indices were found to be less favorable in PCOS women (phenotypes A, B, and C) compared to non-PCOS women. -A significantly higher VAI was observed in phenotype A, and phenotype B was associated with increased BMI.-No difference in adiposity parameters was observed in PCOS women (phenotype D) compared to non-PCOS women. -A significant higher WHtR was observed in PCOS women compared to healthy women.
Zakeri et al. (2024) (30)	PCOS: n = 150, non-PCOS: n = 240	-No statistically significant differences in body composition parameters (fat and lean mass) in PCOS women compared to non-PCOS.PCOS women demonstrated a statistically significant reduction in the fat-to-muscle ratio compared to non-PCOS women.
Noroozzadeh et al. (2022) (23)	Daughters of women with PCOS: n = 211, daughters of women without PCOS: n = 757	Daughters of PCOS women had a 47% higher risk of overweight compared to daughters of non-PCOS women. -No statistically significant difference was observed in the risk of obesity in daughters of PCOS women.
**Others**		
Behboudi-Gandevani et al. (2020) (31)	PCOS: n = 156, non-PCOS (controls): n = 1,304	-PCOS women did not exhibit a heightened risk of developing CKD compared to those without PCOS (HR: 0.91; 95% CI: 0.60, 1.38).
Rostami Dovom et al. (20230 (32)	PCOS: n = 520, eumenorrheic non-hirsute healthy women: n = 1,638	-Occurrence of kidney stone was significantly greater in PCOS women compared to non-PCOS women (12.5% vs. 7.7%), indicating an OR of 1.59 (95% CI: 1.12 - 2.25). - Phenotype A and D were identified as being at particularly increased risk (OR: 1.97; and OR: 3.03, respectively).
Saei Ghare Naz et al. (2024) (33)	1,101 women with recorded PCOS status	-PCOS may not exert an independent effect on hepatic functional parameters.

Abbrreviations: TLGS, Tehran lipid and glucose study; PCOS, polycystic ovary syndrome; NIH, National Institutes of Health; AE, androgen excess; HOMA-IR, homeostatic model assessment for insulin resistance; HOMA-β, HOMA-Beta; IR, insulin resistance; T2DM, type 2 diabetes mellitus; Pre-DM, prediabetes mellitus; HR, hazard ratio; CI, confidence interval; MetS, metabolic syndrome; CVDs, cardiovascular diseases; SBP, systolic blood pressure; DBP, diastolic blood pressure; CAD, coronary artery disease; FRS, Framingham risk score; WC, waist circumference; ABSI, A Body Shape Index; AUC, area under the curve; WHtR, waist-to-height ratio; VAD, visceral adiposity dysfunction; VAI, Visceral Adiposity Index; BMI, Body Mass Index; CKD, chronic kidney disease; OR, odds ratio.

### 3.2. Glucose Disturbances

Early reports from the TLGS participants indicated significant alterations in glucose homeostasis among women with PCOS. In 2015, a study including 85 women with PCOS and 552 control participants without PCOS demonstrated significantly higher levels of insulin, HOMA-IR, and HOMA-Beta (HOMA-β) in women with PCOS (7). Furthermore, longitudinal comparison between the third and first follow-up revealed that insulin and HOMA-IR increased by 6.7% and 14.6%, respectively, in controls, whereas these parameters decreased by 10.6% and 5%, respectively, among women with PCOS (7). This finding was further corroborated one year later by Behboudi-Gandevani, who reported a significantly higher prevalence of IR in women with PCOS compared to those without the condition (34% vs. 26%, P = 0.041) (21). Along with earlier studies that characterized IR in women with PCOS, a subsequent investigation focusing on diabetes-related outcomes further confirmed these findings. A longitudinal analysis conducted by Kazemi Jaliseh et al., involving 178 women with PCOS and 1,524 non-PCOS women, demonstrated that the incidence rates of both T2DM and pre-DM were significantly higher among women with PCOS compared to their non-PCOS counterparts (13.4 vs. 4.2) and (30.3 vs. 23.9), respectively (9).

A more recent publication including 287 women diagnosed with PCOS, 536 women meeting isolated PCOS criteria, and 936 non-PCOS revealed that PCOS women with obesity (either general or central obesity) showed a significantly longitudinal increase in IR in comparison to those without obesity (22). Notably, women with both PCOS and obesity experienced a progressive deterioration in IR, while those with PCOS but without obesity demonstrated an improvement over time. Together, these studies indicate a consistent pattern of IR associated with PCOS within this population.

**Figure 2.**
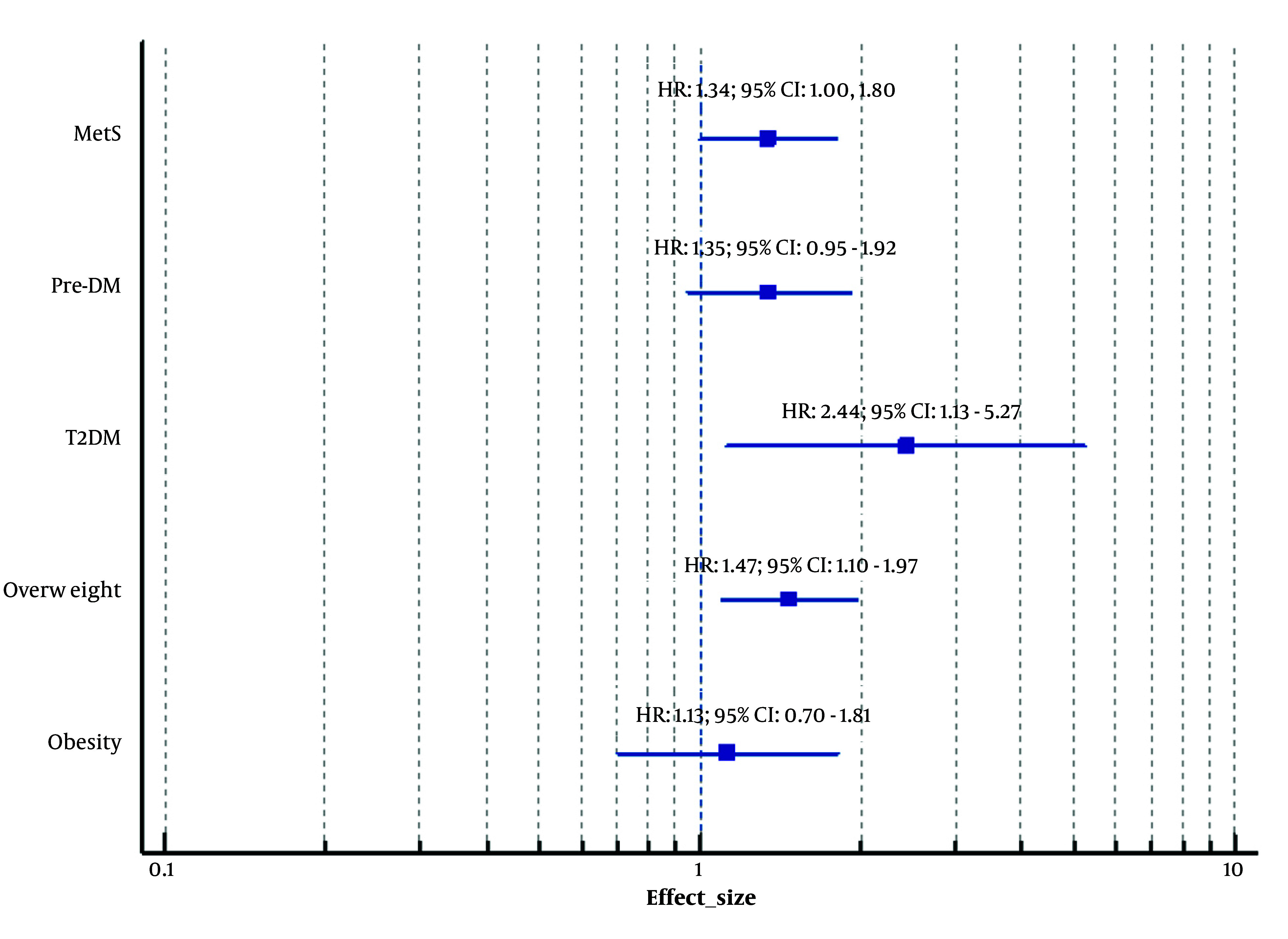
Forest plot: Cardiometabolic disorders in daughters (female offspring) of women with polycystic ovary syndrome (PCOS). T2DM, type 2 diabetes mellitus; Pre-DM, prediabetes; MetS, metabolic syndrome; HR, hazard ratio; CI, confidence interval. Adjusted for potential confounders including Body Mass Index, age, education status, and physical activity.

**Figure 3.**
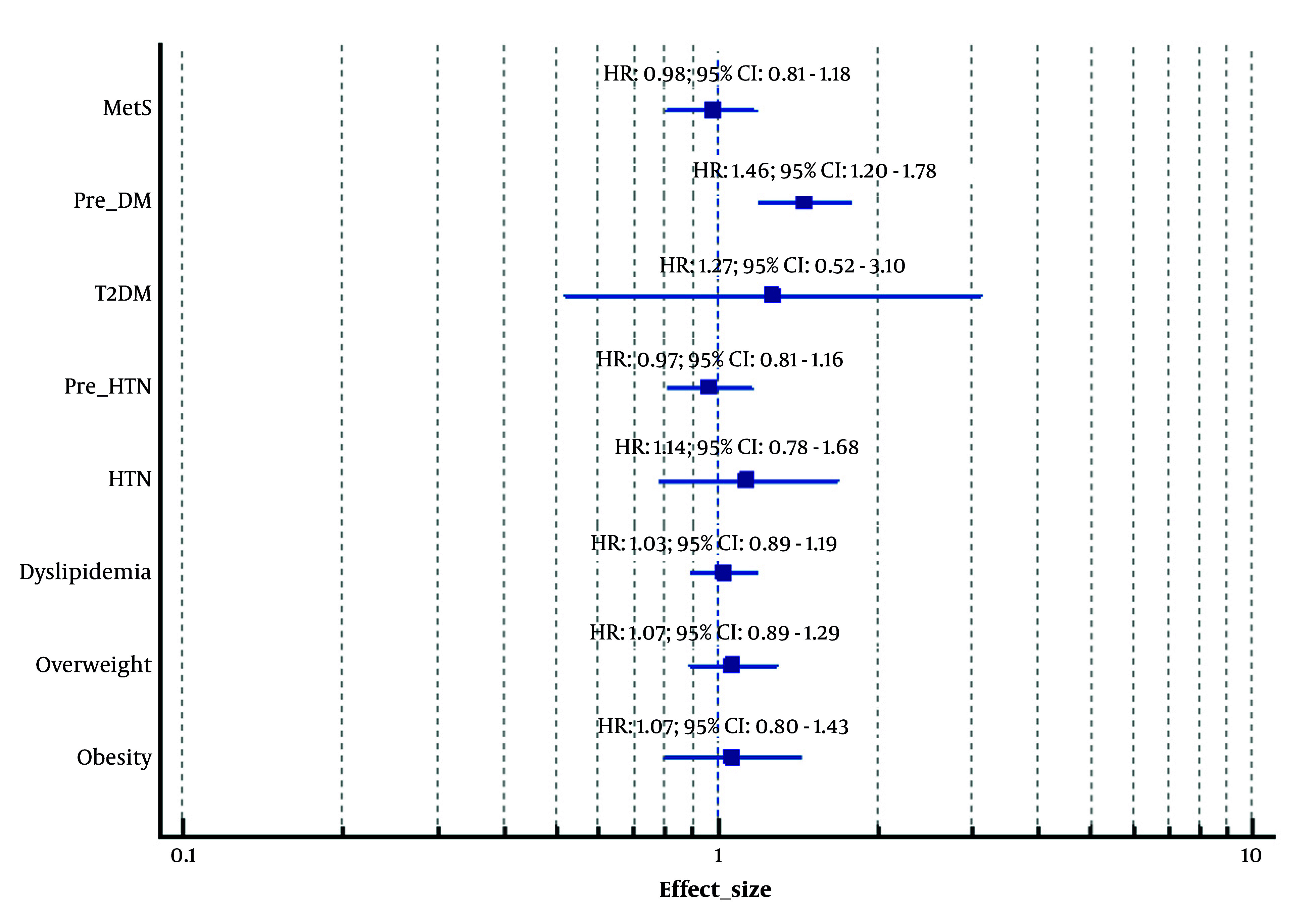
Forest plot. Cardiometabolic disorders in sons (male offspring) of women with polycystic ovary syndrome (PCOS). HTN, hypertension; pre-HTN, prehypertension; T2DM, type 2 diabetes mellitus; Pre-DM, prediabetes; MetS, metabolic syndrome; HR, hazard ratio; CI, confidence interval. Adjusted for potential confounders including Body Mass Index, age, education status, and physical activity.

In addition to the robust evidence of impaired glucose metabolism in women with PCOS, researchers have also investigated the metabolic health of their offspring. A pioneering study conducted in 2022, involving 211 daughters of women with PCOS and 757 daughters of non-PCOS women, demonstrated that the daughters of women with PCOS exhibited a significantly increased risk of developing T2DM, even after adjustment for confounding variables (adjusted HR: 2.44; 95% CI, 1.13 - 5.27) (23). Notably, no increased risk of pre-DM was observed (23). Another study published in 2023, which included 409 sons of women with PCOS and 954 sons of non-PCOS women, demonstrated a significantly higher risk of pre-DM among the sons of women with PCOS following adjustment for confounders (HR: 1.46; 95% CI: 1.20 - 1.78) (24). However, no increased risk of T2DM was observed in this group (24). Figures 2 and 3 and Table 2 present cardiometabolic disorders in offspring.

Targeted preventive interventions aimed at mitigating the elevated risk of glucose metabolism disturbances and related cardiometabolic conditions in this vulnerable population from an early stage may improve long-term health outcomes.

### 3.3. Metabolic Syndrome and Components

Findings on MetS are heterogeneous. The first publication investigating MetS reported no statistically significant differences in MetS prevalence between women with and without PCOS (15% vs. 14%, P = 0.917) (21). In contrast, another study published in Fertility and Sterility in 2018 reported a significant difference in the incidence rates of MetS (21 vs. 22.7) and dyslipidemia (8). Although women with PCOS aged ≤ 40 years had a higher risk of MetS (HR: 1.81; 95% CI: 1.1 - 2.9), this association was not evident in those aged > 40 years (8). Conversely, the risk of dyslipidemia did not differ significantly between the two groups across any age category (8). A recently published analysis of TLGS data in 2022 on PCOS women across different phenotypes revealed that those with hyperandrogenism phenotypes (A, B, and C) exhibited a less favorable lipid profile compared with non-PCOS women (20). In addition, women with PCOS with phenotype A exhibited a significantly higher prevalence of MetS compared to non-PCOS women (20). Women with hyperandrogenism also showed markedly elevated rates of hypertriglyceridemia than non-PCOS women (20). Variations in sample size, follow-up duration, and covariate adjustment are likely to contribute to these observed discrepancies.

In addition to the increased incidence and risk of MetS in women with PCOS, the offspring of those women may also experience such disorders (25). Based on the TLGS dataset, the risk of developing MetS increased in daughters of women with PCOS in their later life before and after adjusting for the potential confounding factors (adjusted HR: 1.34; 95% CI: 1.00 - 1.80), (P = 0.05, borderline) (25). On the other hand, a higher risk of MetS was not observed in the sons of PCOS patients, either before or after adjustment (26). Taken together, these studies suggest that the offspring of women affected by the condition exhibit a greater predisposition to certain metabolic dysfunctions, including abnormalities in glucose metabolism and an elevated risk of developing MetS. Figures 2 and 3 and Table 2 present cardiometabolic disorders in offspring.

### 3.4. Hypertensive Disorders and Cardiovascular Diseases

Blood pressure abnormalities have been extensively investigated within this cohort. In the first publication focusing on women with PCOS from the TLGS population (n = 85), no significant difference was detected in systolic and diastolic blood pressure (SBP and DBP) values when compared with their non-PCOS counterparts (n = 552) (7). These initial findings suggested that, at least in the early phase of follow-up, the presence of PCOS did not independently influence baseline blood pressure parameters. A subsequent publication examining hypertension outcome among women with PCOS reported higher HRs for developing hypertension in those aged ≤ 40 years (HR: 2.08; 95% CI: 1.0 - 3.9) (8). However, this association was not observed among women aged 40 years or older, indicating that the elevated risk was primarily confined to younger age groups (8).

In the first prospective evaluation of CVDs outcome among women with PCOS, conducted on 356 PCOS women and 1,235 non-PCOS participants, no significant association was found between PCOS status and the risk of silent coronary artery disease (CAD) after adjustment for potential confounders (HR: 0.96; 95% CI: 0.86 - 1.08) (27). Regardless of PCOS status, women with a previous history of silent CAD had a 2.25-fold higher incidence of CVDs in later life compared to those without such a history (95% CI: 1.63 - 3.10) (27). Notably, the presence of PCOS was associated with a 42% reduction in CVDs incidence, regardless of silent CAD or conventional risk factors (HR: 0.58; 95% CI: 0.35 - 0.98) (27). These findings suggest that while silent CAD independently contributes to the progression of CVDs, the presence of PCOS may attenuate this risk, potentially through mechanisms related to age distribution, hormonal milieu, or protective metabolic adaptations in younger affected women. Further supporting evidence from Amiri et al. based on a survival analysis of the TLGS dataset revealed that among women with PCOS, each one-unit increase in the Framingham Risk Score (FRS) was associated with a 38% rise in the risk of CVDs (HR: 1.38; 95% CI: 1.14 - 1.66), underscoring the predictive value of conventional risk assessment models in this population (28).

### 3.5. Adiposity

The first publication (2015) assessing adiposity parameters among women with PCOS participating in TLGS reported no significant difference in waist circumference (WC) between PCOS patients and controls (7). In a subsequent study published one year later, other obesity indices were assessed among 704 non-PCOS women and 50 women with PCOS, showing no significant difference in the mean (SD) of a Body Shape Index (ABSI) between the two groups (0.76 (0.05) vs. 0.76 (0.053), P = 0.363) (21). The study also demonstrated that the area under the curve (CI 95%) of waist-to-height ratio (WHtR) for expecting IR and MetS in women with PCOS exceeded 0.75, indicating good predictive performance (21). In contrast, ABSI was not a reliable predictor for these metabolic outcomes.

Another TLGS-based investigation examined the coexistence of PCOS and visceral adiposity dysfunction (VAD), and found that women with both PCOS and VAD exhibited a markedly higher prevalence of cardiometabolic disturbances compared with those without VAD, suggesting a compounding effect of visceral fat accumulation on metabolic risk in PCOS (33).

The study by Behboudi-Gandevani et al. reported that the incidence rates of obesity among women with and without PCOS were 22.6 (95% CI: 15.5 - 33.0) and 24.0 (95% CI: 21.3 - 27.0) per 1,000 person-years, respectively (8). Although the overall incidence rates were comparable, among women aged ≤ 40 years with PCOS, the risk of central and general obesity was notably increased compared to their non-PCOS counterparts (8). In a subsequent cross-sectional analysis of the TLGS dataset (2022), adiposity indices were found to be less favorable in PCOS phenotypes characterized by hyperandrogenism (A, B, and C) than in non-PCOS women (20). Specifically, a significantly higher Visceral Adiposity Index (VAI) was observed in phenotype A [118.8 (60.3 - 213.1) vs. 87.0 (54.9 - 147.1)], whereas phenotype B was associated with increased BMI [26.6 (5.5) vs. 26.2 (4.6) kg/m²] (20). In contrast, phenotype D, defined by the absence of hyperandrogenism, did not differ significantly from non-PCOS women in terms of adiposity parameters (20). Moreover, a significantly higher waist-to-height ratio (WHtR) was observed among women with hyperandrogenic phenotypes compared to healthy women (20).

Recent investigations utilizing data from phase VII of the TLGS have focused on body composition parameters in women with PCOS. The results revealed no statistically significant differences in body composition parameters, such as fat and lean mass, or in additional components assessed by bioelectrical impedance analysis (BIA) between women with and without PCOS (29). Moreover, PCOS status, when evaluated independently, did not exert a significant influence on body composition in both unadjusted and age-adjusted analyses (29). Nevertheless, after adjusting for potential confounders, women with PCOS demonstrated a modest yet statistically significant 0.07% reduction in the fat-to-muscle ratio compared to non-PCOS controls (29).

Extending the assessment to the next generation, a related publication examining the offspring of women with PCOS reported that daughters of affected mothers had a 47% higher likelihood of being overweight compared with those born to non-PCOS women (23). In contrast, there was no statistically significant difference in the risk of obesity across the two groups (23). These findings suggest that while PCOS may have a limited direct effect on body composition in adulthood, its intergenerational influence on weight regulation and adiposity appears noteworthy.

### 3.6. Others

**Figure 4.**
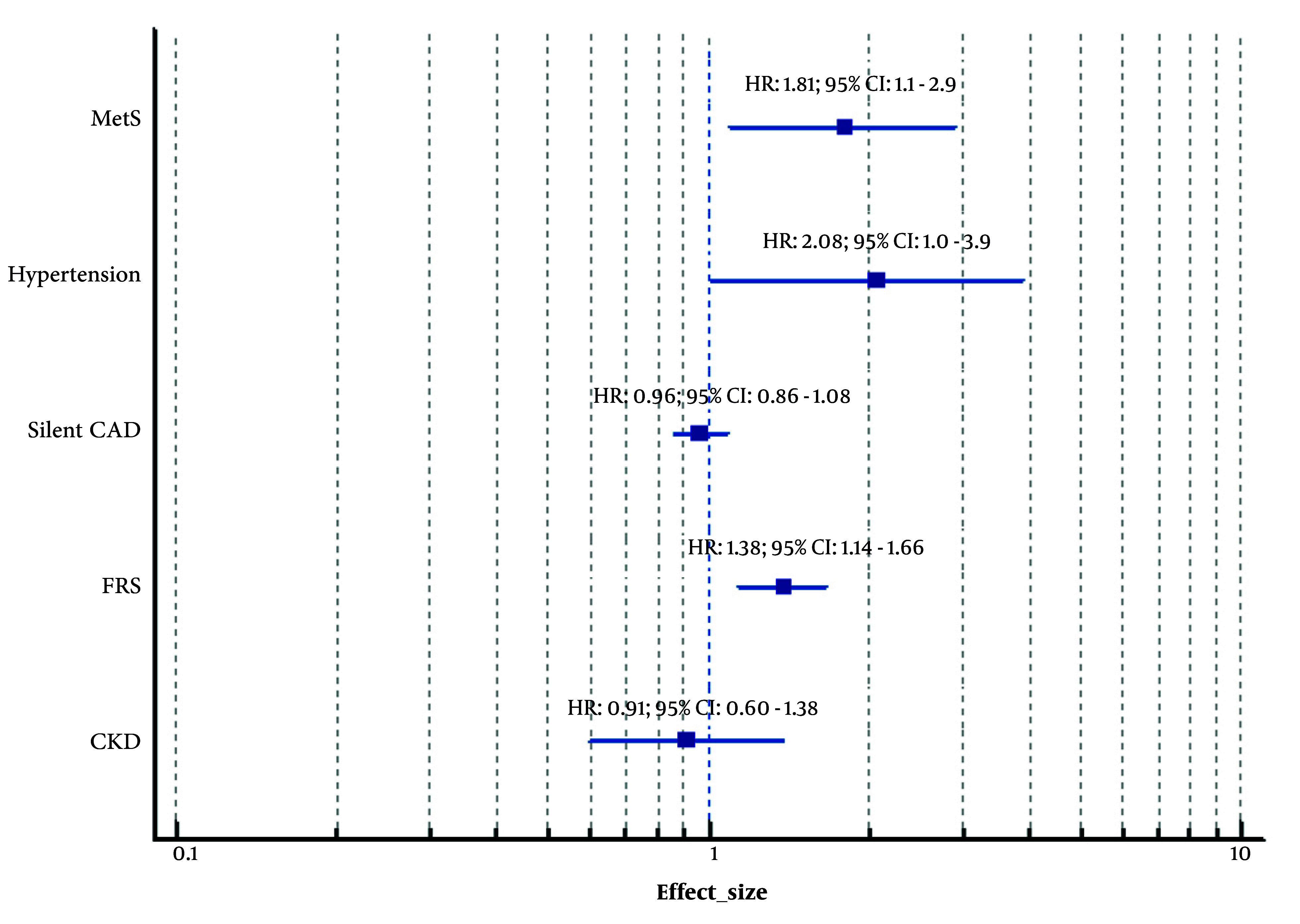
Forest plot: Cardiometabolic disorders in women with polycystic ovary syndrome (PCOS). CKD, chronic kidney disease; FRS, framingham risk score; Silent CAD, silent coronary artery disease; MetS, metabolic syndrome; HR, hazard ratio; CI, confidence interval. Adjusted for potential confounders including Body Mass Index, age, education status, and physical activity.

Analysis of TLGS data demonstrated that women with PCOS did not exhibit a heightened risk of developing CKD compared to those without PCOS (multiple adjusted HR: 0.911; 95% CI: 0.60-1.38; P = 0.66) (30). However, another study suggested that nephrolithiasis may be more prevalent among women with PCOS. Specifically, the occurrence of kidney stones was significantly greater in women with PCOS than in non-PCOS women (12.5% vs. 7.7%, P = 0.001), with an odds ratio (OR) of 1.59 (95% CI: 1.12-2.25, P = 0.01) (31). When stratified by PCOS phenotype, Phenotype A and D were identified as being at particularly increased risk (OR: 1.97, P = 0.02; and OR: 3.03, P = 0.01, respectively) (31). These findings suggest that while overall renal function, as reflected by CKD risk, may not be adversely affected by PCOS, specific phenotypic subgroups could be more susceptible to renal calculi formation. Figure 4 and Table 2 show cardiometabolic disorders in women with PCOS.

Cohort analyses of liver function within the same dataset revealed no significant association between PCOS and elevated liver enzyme levels, indicating that PCOS itself may not exert an independent effect on hepatic functional parameters (32).

### 3.7. Strengths and Limitations of the Present Study

This review synthesizes findings from a well-established, high-quality cohort study, providing access to prospectively collected data with standardized methods across all publications. The cohort design minimizes recall bias and ensures temporal clarity between exposure (PCOS) and cardiometabolic outcomes. Another strength of our study is contributing valuable insight into cardiometabolic health in women with PCOS and the next generation. Moreover, given that TLGS is a unique cohort with over two decades of follow-up in a population representative of the capital city of Iran, it can offer an important overview of PCOS and its impact on women’s and family health in the Iranian population. No other Iranian study provides comparable long-term data. Conversely, our study also has several limitations that should be acknowledged. All included papers originate from a single cohort and from an urban Iranian population, which may limit generalizability to other populations with different demographic or healthcare characteristics. Another limitation is the lack of data on cardiometabolic health of women with PCOS during menopausal ages. Additionally, lifestyle modifications such as dietary habits were not measured in women with PCOS who participated in TLGS, which potentially may influence adverse cardiometabolic outcomes.

## 4. Conclusions

The observed heterogeneity in cardiometabolic outcomes across age groups and PCOS phenotypes underscores the importance of implementing age-specific and phenotype-directed metabolic screening protocols. In younger women, heightened vigilance for obesity, central adiposity, and lipid abnormalities may enable timely interventions. In contrast, in older women, attention should focus on monitoring cardiovascular risk factors that may manifest later in life. Furthermore, the evidence pointing to increased overweight and T2DM risks in daughters of women with PCOS calls for early lifestyle counseling and preventive strategies targeting childhood and adolescence. Given the complexity of PCOS pathophysiology and its broad systemic effects, long-term prospective studies are essential to elucidate causal pathways linking reproductive and metabolic dysfunctions and to identify modifiable targets for intervention. Such studies should integrate hormonal, genetic, and lifestyle factors, and assess how tailored strategies, ranging from dietary modification and physical activity to pharmacological therapies, can mitigate the cardiometabolic burden associated with PCOS. Ultimately, a comprehensive, lifespan-oriented approach is warranted to reduce the intergenerational transmission of metabolic risk and improve health outcomes for both women with PCOS and their offspring.
